# Development of an mHealth Platform for Adolescent Obesity Prevention: User-Centered Design Approach

**DOI:** 10.3390/ijerph191912568

**Published:** 2022-10-01

**Authors:** Catarina I. Reis, Cláudia Pernencar, Marta Carvalho, Pedro Gaspar, Ricardo Martinho, Roberta Frontini, Rodrigo Alves, Pedro Sousa

**Affiliations:** 1 ciTechCare—Center for Innovative Care and Health Technology, Polytechnic of Leiria, 2410-541 Leiria, Portugal; 2 Arts and Design Research Lab (LIDA), Polytechnic of Leiria, 2411-901 Leiria, Portugal; 3 NOVA Institute of Communication (ICNOVA), Nova School of Social Sciences and Humanities, 1069-061 Lisboa, Portugal; 4 School of Technology and Management, Polytechnic of Leiria, 2411-901 Leiria, Portugal; 5 CINTESIS, University of Porto, 4099-002 Porto, Portugal; 6 CIEQV—Life Quality Research Centre, Polytechnic of Leiria, 2411-901 Leiria, Portugal; 7 Health Sciences Research Unit: Nursing (UICISA: E), Nursing School of Coimbra (ESEnfC), 3004-011 Coimbra, Portugal

**Keywords:** obesity, adolescents, mHealth, user-centered design approach, design process, PSSUQ

## Abstract

Obesity is a chronic condition that influences the quality of life of patients and families while increasing the economic burden for the world population. Multidisciplinary prevention programs are crucial to address it, allowing an early introduction of healthy behaviors into daily habits. Mobile health interventions provide adequate support for these programs, especially considering the gamification techniques used to promote users’ engagement. TeenPower is a multidisciplinary mHealth intervention program conducted in Portugal during 2018 to empower adolescents, promoting healthy behaviors while preventing obesity. An agile software development process was applied to the development of the digital platform that holds a web-based application and a mobile application. We also propose a model for future developments based on the user-centered design approach adopted for this development and the assessment conducted in each phase. The user-centered design approach model proposed has three distinct phases: (1) design study; (2) pre-production usability tests; and (3) post-production data. Phase 1 allowed us to obtain the high-fidelity version of the graphical user interfaces (*n* = 5). Phase 2 showed a task completion success rate of 100% (*n* = 5). Phase 3 was derived from statistical analysis of the usage of the platform by real end users (*n* = 90). We achieved an average retention rate of 35% (31 out of 90 participants). Each technique has provided input for the continuous design and improvement of the platform. This allowed the creation of a tailored platform that could meet users’ expectations. Nevertheless, the retention rate decreased significantly over a short period of time, revealing the need for further work in the improvement of the gamification experience.

## 1. Introduction

### 1.1. Context

Obesity is a serious chronic health condition, considered a public health problem [1,2,3,4] with epidemical levels worldwide [5] and increasing prevalence in adolescence, including in Portugal [6,7]. It can lead to the development of physical and psychosocial complications such as lower levels of quality of life and higher levels of psychopathological symptoms [8,9,10,11,12] and is linked to an increase in the economic burden in the near future [13,14]. Considering the aforementioned consequences related to this condition, the prevention of youth obesity is of utmost importance and should be a priority [15,16]. Considering that the etiology of obesity is multifactorial [5,17], prevention programs should be multidisciplinary, and integrate innovative components such as mHealth strategies.

mHealth interventions have emerged due to the ubiquity use of mobile devices, particularly smartphones, and were leveraged by the fact that mobile phone users carry them around constantly, making it possible to deliver meaningful health information as users go about their everyday activities [18,19,20,21,22,23,24,25,26,27,28,29,30,31]. They improve the early detection of medical complications and might help in the prevention of unnecessary hospitalizations [30]. In this prevention context, they are an optimal vehicle to allow the design and implementation of timely interventions based on user behavior, such as those related to obesity [32].

When considering best practices for multidisciplinary interventions, direct associations have been found between treatment intensity and involvement, and meaningful clinical outcomes [33]. The mobile nature of mHealth apps allows them to be used in multiple environments. This could enhance the effectiveness of the intervention and have a major role in the adherence to treatment, promoting self-monitoring, and feedback. 

### 1.2. Related Work

User-centered design (UCD) is widely known and there are several models that provide an actual implementation to the approach [34,35,36,37]. “User-centered design (UCD) is an iterative design process in which designers focus on the users and their needs in each phase of the design process. In UCD, design teams involve users throughout the design process via a variety of research and design techniques, to create highly usable and accessible products for them” [38]. This clear, currently available definition of UCD is an evolution of the initial approach proposed by Norman in 1986 [34]. UCD models focus on human–computer interaction (HCI) procedures jointly with constant feedback of the users that is welcomed and used to improve the system. All these models provide an orientation about the phases and workflow to be conducted with the users or stakeholders of the system. Only one of the approaches is completely health-related and includes a reference to patient-centered design (PCD) [36]. None of the models provides actual guidance of the usability methods to be used in each phase. This is of major relevance as is shown by a recent systematic guidance that addresses the need to recommend usability methods for specific project’s stages and constraints [39].

Interactivity is an essential feature in e-programs for adolescents [40,41] and mHealth systems are using a rich user experience that includes games to engage adolescents [42,43,44], improve health outcomes and empower behavior change [45,46,47,48]. Recent studies [49,50] underline the growing use of gamification and serious gaming in health and wellness contexts that improve self-management behaviors. 

Methodologies from practitioners and researchers in the HCI field include adding specific gamification elements [42,47,51,52,53] such as badges, leaderboards, ranks, enduring play, levels, self-representation with avatars, three-dimensional environments, narrative context, feedback, competition under rules, teams, parallel communication systems that can be easily designed, and time pressure [54]. Nevertheless, existing studies [47] do not present preliminary results of how: (1) interface design patterns; (2) game design patterns or game mechanics; (3) design principles, heuristics or ‘lenses’; (4) conceptual models of game design units; (5) game design methods and design processes; are applied in games for health contexts. Thus, there is a need to better understand the game’s elements significance in the health context, pairing game design and health concepts to identify the corresponding universal principles of design, such as storytelling, affordance, consistency, and mental model [55,56].

An interesting study related to serious game design shows that evaluating user experience helps to identify aspects in the game mechanics (e.g., the most appropriate device, continuous feedback, challenges, scoring evaluating user experience system, and learning levels) while providing guidelines to help both therapists and teachers to achieve pedagogical objectives [57].

Regarding the specificity of the topic of prevention of adolescent obesity, we explored applications that were available to the general public [58,59,60]. Most applications have auto-monitoring features (to be used by adolescents) that allow adults (teachers, health professionals) to oversee the progress related to food intake, rest/sleep habits, and exercise. Nevertheless, an in-depth exploration of the applications showed that only one of them had built-in games, only one other one had daily challenges, and none had leaderboards nor social interaction modules. None of the applications explored seemed to have the important concept of “designed like a game” present.

A recent literature review provides a list of studies where adolescents would self-monitor and follow dietary and exercise suggestions using mHealth applications [33]. The results obtained in the studies are heterogeneous and two important needs were raised: (1) address the engagement with mHealth apps and (2) usability measurement tools for use in research and practice of mHealth interventions for childhood obesity treatment.

A limitation related to current mHealth interventions is app underutilization. A recent study carried out with more than 318,000 health apps and 340 consumer wearables (and with over 200 health apps being added to the market each day), suggested that most apps are underutilized [61]. A quarter of all app downloads are used only once, and later statistics revealed a user retention rate of 32% for the first months of 2019, with a 71% churn rate (users that returned to the application eleven times or more) [62]. Consumers, especially adolescents, often tend to not return to applications that do not immediately engage them, therefore undermining interventions’ potential effectiveness [63,64,65,66]. 

Therefore, it is crucial to design and develop applications using a user-centered design (UCD) approach that engages users from the start [34,35,36,37].

### 1.3. Goal

TeenPower provides an mHealth platform for the prevention of obesity in adolescents. A UCD agile software development process was adopted with an iterative and incremental approach for designing and implementing the platform in a permanent dialogue with healthcare professionals and adolescents. Our focus is to verify if the usage of a three-phase UCD approach to design and develop an mHealth app for preventing adolescent obesity provides a higher user retention rate when compared to the reported retention rates of similar apps. Our approach is mainly qualitative since our goal is to establish recommendations to be used to conduct similar platform developments in the future. The work here described presents the theoretical rationale, design, and preliminary results achieved.

## 2. Materials and Methods

The TeenPower e-therapeutic digital platform was developed by a multidisciplinary team and includes a back office and a mobile application [67]. The back office is a web-based application created for teachers and health professionals to support the decision-making process regarding the customization of the mHealth intervention. It includes several features: user and educational content management (e.g., videos, images, documents), social interaction (e.g., private chat and discussion forums), and data analysis with interactive charts and filters (dashboard with all the users’ monitoring data such as physical condition, eating habits, hydration, physical activity, and sleep monitoring). 

The mobile app is developed for the Android platform and is directed at adolescents. It includes educational resources, self-monitoring features (such as eating habits and hydration), chats, discussion forums, personalized messages, and motivational tools (such as progression of health behaviors and biometric data, positive reinforcement). It seeks to create a virtual environment attractive to adolescents, with a game-based learning process, where their engagement is rewarded with points and coins, progressing in the hall of fame. The purpose is to affect their behavior by allowing them to experience non-recreational purposes, focusing on areas such as physical exercises and nutrition habits. The engagement is defined to occur from the moment that each user begins their interaction with the mobile app with the opportunity to customize her/his avatar. The avatar status is incremented and updated whenever adolescents complete a suggested task (such as registering their eating, drinking, and resting habits). All this information is closely supervised by the health professionals that are tracking the adolescent’s behavior through the back office application. 

### 2.1. Three-Phase UCD Model

The TeenPower platform was developed using an UCD model, where three distinct evaluation phases were identified.

**Figure 1:**
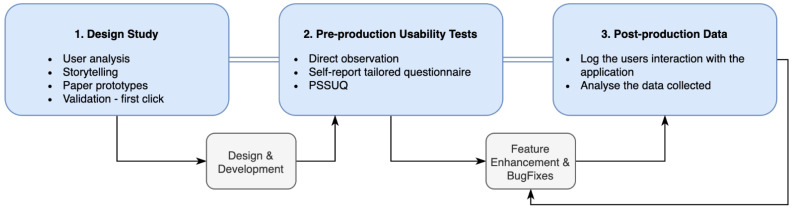
Three-phase user-centered approach model—techniques and method details.

Figure 1 presents the user-centered approach conducted through the three evaluation phases where the interaction with users occurred. For each phase, a custom evaluation method was used, which allowed us to adjust the platform’s development while maximizing the feedback given by users.

The methods used in each phase were different and adjusted to the development stage of the platform. The metrics involved in each method are not comparable per se, but the main goal is to have feedback to incorporate in the development of the platform.

#### 2.1.1. Phase 1—Design Study

The methods used in Phase 1 (design study) fall directly into the six phases of the design process and were chosen to allow us to obtain the high-fidelity version of the graphical interfaces. Addressing the problem of combining a gamification technique with the design process is a challenge. Research studies [42,44,52] show that the design field is more open now to a deep understanding of human motivation. So, it is believed that designers recognize how to match game elements that make sense to users with principles of design [55]. Creating clear connections to a given activity and supporting feelings of autonomy are identified as essential issues in self-determination theory, which emphasizes the meaningfulness definition inside of the design process [68]. On the contrary, non-meaningful elements may be ignored by the users or may demotivate them [52,69]. Our work will identify the elements of design that promote autonomy and relatedness, two of the three fundamental needs presented by SDT. These are the elements that will be considered meaningful for the design process and that will ultimately be implemented in the platform. The design process goes through different phases, obtaining relevant feedback from stakeholders, while iteratively and incrementally increasing the product effectiveness and usability. Several authors [53,70,71,72,73] argue that it should include, mainly, six phases, and the preliminary design study phase included some of them. 

First, a user analysis was conducted [74] to understand the profiles to be considered while designing the platform. “For the purpose of this paper, a user is defined as (a) a person who causes a decision or information system to act or serve a purpose, (b) a person who brings a decision or information system into service or (c) a person who avails himself of a decision or information system” [75]. From this definition, TeenPower user profiles were identified, and the elicitation of requirements started for each one. These findings led us to the creation of an mHealth experience that could address the users’ needs [76]. 

We then conducted two other phases, (1) user journey—a storytelling technique to identify specific elements of features that should be included in the platform; and (2) paper prototype technique [77]—to accomplish the initial definition of ideas for the features that cater to users’ expectations and to comprehend how the visuals match. Finally, a validation of the paper prototype was conducted using the first click method [78]. 

Phase 1 of the study involved five participants from a secondary school based in Leiria, Portugal (*n* = 5) that were recruited during an initial presentation of the project [79]. The students group consisted of two girls and three boys with an average age of 15 years (SD = 1.4). The goal of this preliminary study—usability tests in paper prototype—was to validate initial user experience assumptions with a small sample size. Other design methods such as a user journey and personas were previously followed [79,80]. Results of these processes come together within research team decisions.

#### 2.1.2. Phase 2—Pre-Production Usability Tests

The second phase of the approach focused on the usability testing of the developed applications [81,82]. Periodic meetings were held with the various user profiles (teenagers, health professionals, and teachers) who were involved in the development process, gathering contributions on the necessary features and possible gamification components. Two usability sessions were conducted in this phase: (1) the mobile application session; and (2) the web back office application session. The TeenPower multidisciplinary team volunteered to conduct these tests. Only research team members who did not have any previous contact with the applications served as volunteer testers. This ensured that behaviors observed and registered during each test session would resemble those of an audience during their first interaction with the applications. The group was split into three different roles: volunteers, interviewers, and observers. This amounted to a total of nine volunteers (*n* = 9), mostly female participants (67%, 6 out of 9) and, on average, aged 48 years old (SD = 9.6).

The structure of the first session relied heavily on direct observation in which all behaviors, actions, and reactions of the user were observed and recorded before, during, and after performing a certain predefined task in the application [75,77]. The session followed a hands-on interview, using a previously prepared script that contained a series of questions, purposely crafted to guide users into testing certain features of the application. The script proposed three challenges to users (see Appendix A). Upon the conclusion of the interviews, volunteers freely explored the application and answered a self-report questionnaire to voice their opinion regarding their experience with the available navigation flow and the perceived ease of use (see Appendix B). During the mobile app session, 3 pairs of interviewers + observers conducted a direct observation (DO) process on 7 volunteers (*n* = 7). Two other volunteers started exploring the application autonomously (*n* = 9).

For the web back office application session, all the volunteers (*n* = 9) were given a twenty-minute time-box in which they had full access to the application and could freely explore it. A time-box established a common ground for all the participants in the evaluation—they had the same amount of time to explore the features of the back office, which was completely unknown to them. Simultaneously, observers took note of defects and facilitated those who became stuck in some feature. At the end of the session, the validated Portuguese version of the post-study system usability questionnaire (PSSUQ) [83]—a 19-item instrument—was handed out to quantify the overall system usability and, specifically, the quality of the information (InfoQual sub-score), the quality of the system, and the quality of the interface (SysUse and IntQual sub-scores). In the PSSUQ, lower scores indicate better results and, if an item is answered with N/A, the score of such an item is the overall average of the remaining items (further detail is available in Appendix C).

Thus, the methods used in Phase 2 join the direct observation method, with a custom-made questionnaire and a standard validated questionnaire (PSSUQ).

#### 2.1.3. Phase 3—Post-Production Data

Both the mobile and the back office applications are available to actual users in a real environment. The focus of Phase 3 is on the actual usage of the platform by real end users. Overall, 90 users used the app in the period under study and no further recruitment has been conducted after the controlled trial occurred [84].

Google Analytics for Firebase [85] is a free analytics solution that integrates with existing applications and provides useful insights on data collected. This platform allows an extra layer of services to overview and visualizes the actual behavior of the application and of the end users’ actual usage of the application (end-user engagement). Crashlytics (and crash reporting) is a service of Google Analytics for Firebase that logs events for each crash of the application, allowing the development team to better provide help to the users of the application. The dashboard allows you to visualize the data, filtering it and adapting the queries to understand the actual usage of the application. 

We made a connection to analytics to keep the development team updated on crashes and bugs when they happen in real time, and to analyze information regarding the actual usage of the mobile application.

## 3. Results

### 3.1. Phase 1—Design Study

User analysis allowed the identification of three profiles: adolescent, health professional, and teacher.

The storytelling technique allowed us to pinpoint: (1) the character of the game—representing the adolescent involved in the story; and (2) the narrative—helping to understand the sequence of the events that should occur in the mobile app.

Regarding what should be included when designing the high-fidelity interfaces, the results of the validation showed that four out of five participants (80%) felt distinct difficulties with the option “feed the character”. Further questioning of the participants revealed that the perceived difficulties were mainly because of some graphic elements not being visually well defined. The feedback collected also pointed out that most of the adolescents’ suggestions were based on contributions to improve the game, and not on the existing relationship of doing healthy activities when they are having fun.

Based on these major findings regarding the mobile app study, we paired game elements to the corresponding universal principles of design [55] and the corresponding actual layout of concepts/procedures of the application (represented in the Figure 2, Figure 3, Figure 4, Figure 5, Figure 6, Figure 7, Figure 8, Figure 9 and Figure 10 below), in Table 1.

The strategy presented in Table 1 shows how game elements [56] in mobile apps could be used to define health procedures. In this particular case, the design team applied the universal principles of design [55] justifying their decisions. These principles follow visual examples of concepts applied in practice.

The web back office application prototype was also validated in the same session by two experts (a health professional and a teacher), allowing us to elicit additional functional requirements not previously considered. However, due to the sample size being so reduced (*n* = 2), the results were considered not significant, and we decided to not include them here.

### 3.2. Phase 2—Pre-Production Usability Tests

**Figure 11:**
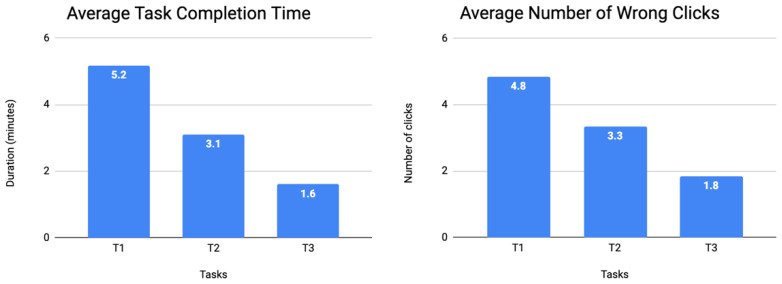
Average task completion time and average number of wrong clicks for the DO process.

During the DO process, we registered an overall average completion time of the proposed tasks of 3.3 min (SD = 1.8) and an average number of 3.3 wrong clicks (SD = 1.5). Figure 11 presents the average results per proposed task. During the mobile app session, two volunteers owned and used iPhones daily, and that, as will be explained later, could be a major reason for some misbehaviors.

**Figure 12:**
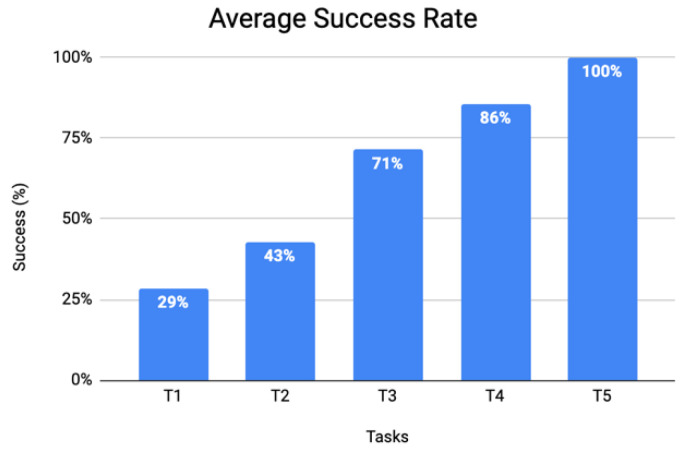
Average success rate for the mobile application usability session.

There is a decrease in the average time spent and in the number of wrong clicks regarding the completion of a task, as the user grows more familiar with the application (Figure 11). This is supported by the results of the success rate (Figure 12) that show that after completing the first two tasks, 71% of the users (six out of nine) successfully completed the third, and 100% of the users were able to successfully complete the last task.

The self-report questionnaire (SRQ) results showed that users spent an average of 17 min to explore the application before finding it easy to use (SD = 7). One out of nine users reported that, even after 30 min of exploration, s/he still felt like there were more content and features to explore, while another user (out of nine) referred that there was a learning curve that had to be mastered before the application became easy to use.

Most of the data collected in the DO and SRQ are qualitative. Thus, specific procedures and care should be taken when analyzing the results [86,87,88,89]. All the free format answers that were given both by observers or volunteers were analyzed and we created categories to group the occurrences and incidents reported. An occurrence means that a participant explicitly did and/or stated something that is included in a category.

Three major categories were identified:

- Avatar (A)—six (out of nine) occurrences

Issue: the option to update the profile image was not available if users pressed the avatar image on the home screen (confusion with profile image). Participants reported that simply discovering and updating the avatar was a challenging, although feasible feature. 

Result: The avatar is, now and by default, distinct from the one used in that same user’s profile;

- Information (I)—six (out of nine) occurrences

Issues: Users promptly dismissed the initial set of balloons with the tips that serve as guidance for the first-time interaction with the application. Most users dismissed the tips without reading them. It was not possible to replay those balloons and tips. Users also experienced some difficulties while trying to register their food intakes by using the available “food-wheel” (an object that mimics the health pyramid).

Result: The tip balloons destined for first-time usages can now be accessed at any time, even after their dismissal. Error messages were also updated so that the user could obtain more useful information regarding the usage of the “food-wheel”;

- Navigation Flow (NF)—five (out of nine) occurrences

Issues: While trying to navigate throughout the screens of the application, users felt the need to know their current location inside the game (breadcrumbs). It was not clear that every time a user needed to do something different, they should first return to the map. iPhone users showed some difficulties regarding the navigation flow in Android, namely with the existence of a back button, distinct from the back-toolbar button offered in iOS applications.

Result: The application currently provides information on the current position of the user (e.g., kitchen, bedroom, gym, …) and includes new visual aids such as arrows and element borders/highlights to provide further guidance in the navigation flow of the application.

**Figure 13:**
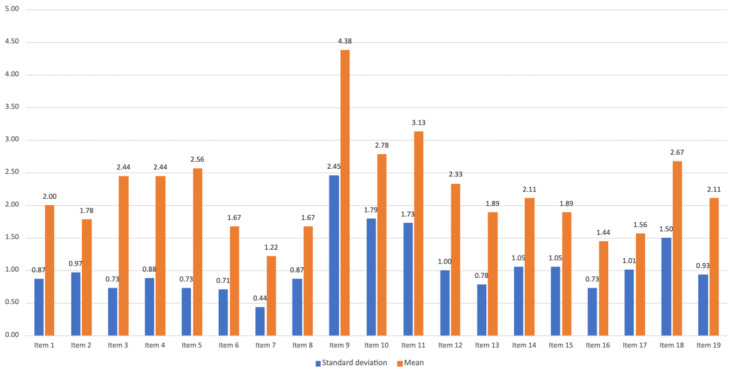
PSSUQ items average scores.

Regarding the PSSUQ evaluation of the web back office application, Figure 13 presents the average score on each item after using the application in a free usage format. Item 7 (“It was easy to learn to use this system”) recorded the lowest average with 1.22 (SD = 0.44), and item 9 (“The system gave error messages that clearly told me how to fix problems”) recorded the highest average with 4.38 (SD = 2.45). 

Looking deeper into these items and the meaning of their score, item 7 refers to the ease of usage of the system, which means that participants globally considered the system to be easy to learn to use. Only one tester reported that it implied a small learning curve. Item 9 refers to the support that the system offered when an error occurs. Thus, the high score means that when errors occurred, users had difficulties to solve the problem and the system feedback did not present a clear path to a solution.

**Figure 14:**
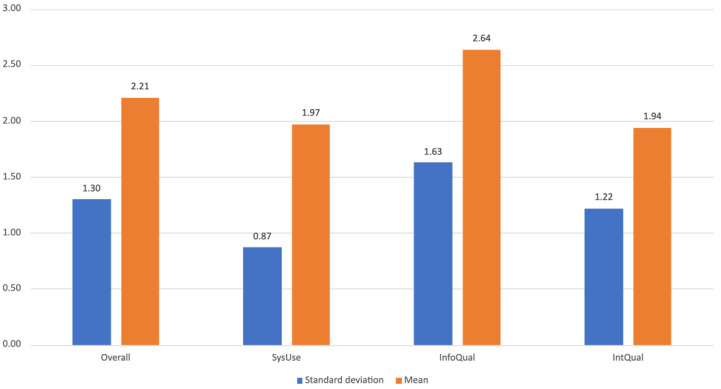
PSSUQ Overall, SysUse, InfoQual, and IntQual scores.

Figure 14 shows the aggregated results from the PSSUQ that show both the system use as well as the interface quality as very good, with the average score on such areas being slightly below 2.0 (1.97 (SD = 0.87) and 1.94 (SD = 1.22)). Information quality was considered the area in which the system requires most improvements, with an average score above 2.5 (2.64 (SD = 1.63)).

### 3.3. Phase 3—Post-Production Data

**Figure 15:**
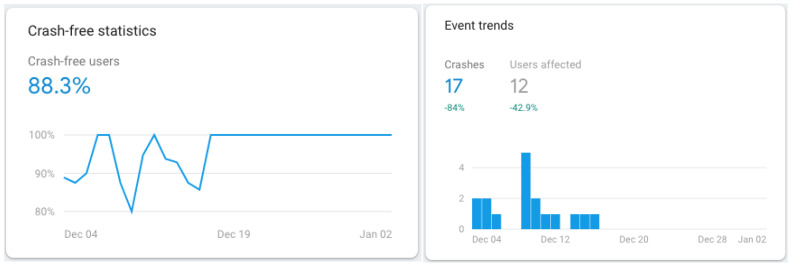
TeenPower mobile app—crash-free statistics (30-day period starting on 4 December 2018).

We consider that our mobile application is stable since 17 December 2018 with a report of 100% crash-free users since that date and until 2 January 2019 (see Figure 15).

**Figure 16:**
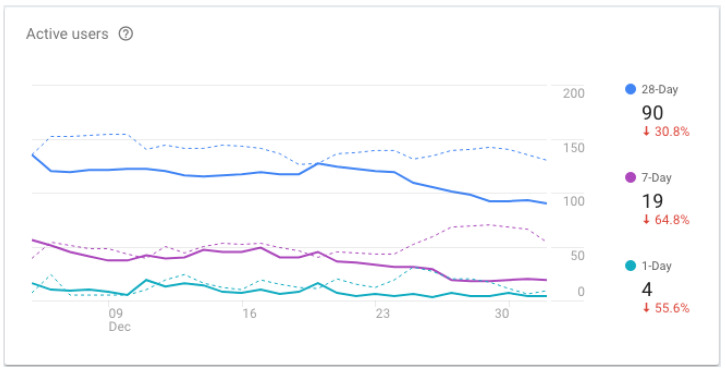
TeenPower mobile app—active users (30-day period starting on December 4, 2018).

Figure 16 presents the number of active users of the application for a 30-day period. This number is based on an actual engagement with the app in the foreground of the device. An average of 4 users are using the app on a daily basis, 19 users are using the app on a weekly basis, and overall, 90 users have used the app in the previous 30-day period.

**Figure 17:**
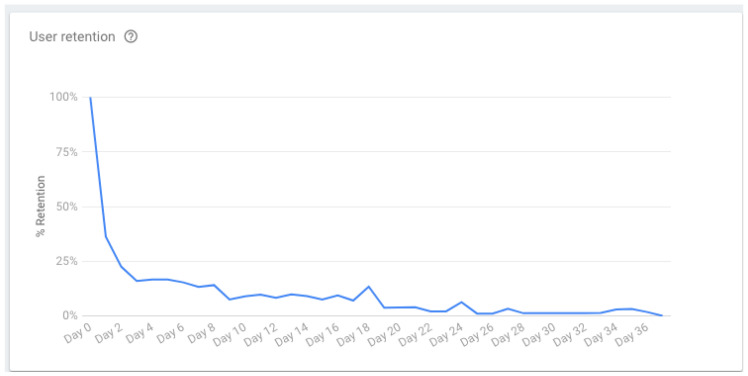
TeenPower mobile app—user retention (from 11 November to 29 December).

The retention rate dropped along a 36 day-period that started on 11 November (Figure 17). On the second day, the immediate user retention rate had already dropped below 25% (less than 22 users out of 90). The rate illustrates two peaks that are ultimately related to intervention kickoff sessions to present the mobile application to new sets of users. The last value registered for the user retention rate was nearly 2% (2 users out of 90). 

**Figure 18:**
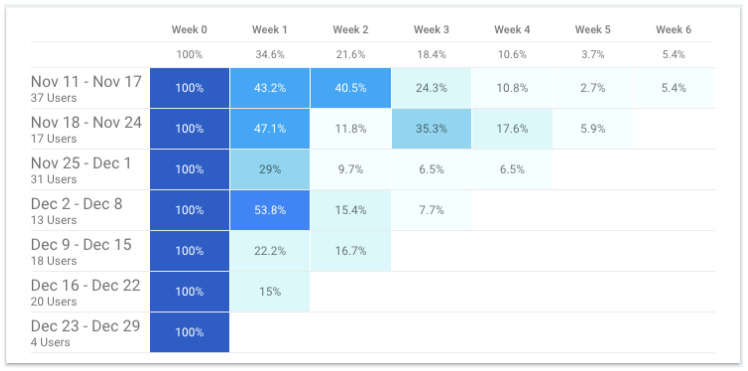
TeenPower mobile app—cohort report (from 11 November to 29 December)—the different shades of blue provide visual clues regarding the intensity or engagement of the users – a darker shade will imply a higher percentage. For instance, from all the users that started using the application in the week from November 18 to 24 (100%) only 35.3% used the application 4 weeks after (Week 3).

The number of users that started using the application in the week of 11 December was 37 and only approximately 5% (2 users out of 37) of these users were still engaged with the app six weeks later (Figure 18). Only nearly 7% (2 users out of 31) of users that started using the application in the week of 25 November were still engaged after four weeks. Nevertheless, for the first few weeks after the initial usage of the application, there was an average retention rate of 35% (31 users out of 90).

## 4. Discussion

Phase 1 of the study allowed us to have a deep insight into the user profiles and the requirements that led to the development of the features currently available in the mHealth platform. The early identification of three distinct profiles allowed us to tune in the design approach. What has started as a patient-centered design (PCD) approach considering adolescents as the only users of the platform was early revised to become a user-centered design (UCD) approach [36]. Our work also considers the roles of health professionals and teachers, which are crucial for the effectiveness of the intervention [84]. Furthermore, the major findings regarding the mobile app study conducted over storytelling and in paper prototype provided a mapping of game elements to the universal principles of design and the corresponding actual layout of concepts/procedures of the application. Moreover, it also contributes to the important “designed like a game” concept. This can be used as a basis for the development of further applications and contributes to a much-needed understanding of the application of game elements in a health context [62] and serious game design [57].

The Phase 2 results show that the time spent in the exploration and actual usage of the application increases the ease of the interactions with the system. The initial design with all the game elements (result from Phase 1) was validated. The participants easily learned the gamification strategy present in the mobile app, and the issues raised with the avatar (game element) were promptly solved. All the information and navigation flow issues were not directly connected to the gamification of the application and were also adequately solved. This phase allowed us to also obtain qualitative feedback and improve both the mobile app as well as the web back office application.

Phase 3 assessed generic user engagement to the platform. The non-intrusive collection of usage data for the mobile and web applications of the platform provided evidence that Crashlytics from Google Analytics, or other similar tools, might find useful to complement existing metrics.

Overall, the three-phase UCD model that we propose describes, for each phase, the methods, metrics, and tools that can be used for the development of an mHealth platform. We also showcase how we applied them to the development of an actual platform centered on its users: TeenPower. This is a step forward in the already identified need to recommend usability methods for a specific project’s stages and constraints [37]. Furthermore, the continuous iterations and improvements that the model promotes allowed us to have a platform in production and available to be used [84].

### Limitations

We strongly believe that the iterative development approach, totally user-centered, allowed a reported retention rate of 35% for the first few weeks. Despite not being the optimal value that we aimed for, it is actually above the overall retention rate (32%) for mobile apps [4].

Furthermore, a retention rate that severely dropped in a few weeks was attributed to the short time span of the intervention [84] and is aligned with the results of several previous studies [4,33,62]. This led us to consider revising the approach for the next interventions. TeenPower was integrated into specific courses held with specialized personnel. Once the classes of these courses ended, the mobile app’ dropout rate increased exponentially, and the adolescents demonstrated their lack of further engagement. Specific motivational and engaging actions that include improving the gamification aspects of the platform and personal interaction with prizes and achievement recognition should be taken to accomplish an actual engagement of adolescents using the mobile application.

The mean age of the volunteers in Phase 2 highly deviates from the target group of expected users of the mobile application. Nevertheless, this was carefully considered since we opted to conduct information quality validation and assessment throughout, besides the usability assessment. Several studies show that while there might be several differences between younger users and older users, a mean age under 50 years old can still be classified as “younger adults” while providing mixed usability results [5]. Furthermore, principles such as feasibility data and benefits, among others, should also be considered for these evaluations [6].

The third phase of our UCD model lacks further observation that is only possible by dedicating enough time to observe the impact of providing additional features to the existing applications.

## 5. Conclusions

The three-phase UCD model provides a reference and suggests feedback methods for each phase. This model can be used in the future by software developers when conceiving similar platforms. 

Each method included in the model provided significant input to use as feedback for the continuous design and development of the platform. Its usage allowed us to develop a quality product tailored to the users by engaging them since the beginning of the development lifecycle, and thus delivering a product with an initial high engagement rate. The iterative development approach, totally user-centered, has allowed a reported retention rate of 35% for the first few weeks. Nevertheless, and since the retention rate decreased significantly over a short period of time, there is a need for further work in the development of a true gamification experience that better retains and engages users for longer periods of time. We aim to be able to conduct such a longitudinal study in the near future.

Since the metrics for each method are not comparable per se, one additional insight could be achieved by applying this model to other contexts, thus allowing an actual comparison: phase by phase, method by method, and metric by metric.

## Figures and Tables

**Figure 2:**
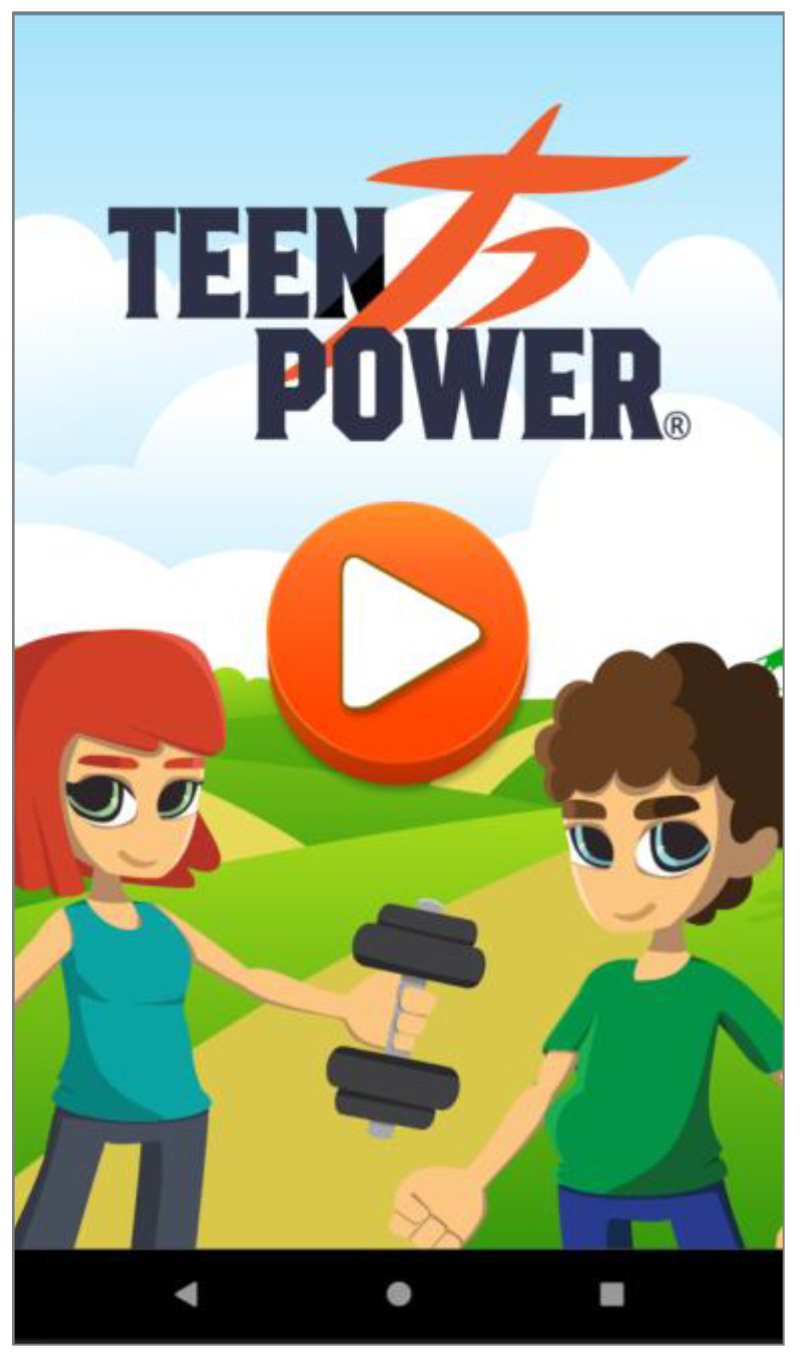
Home screen—affordance; interaction.

**Figure 3:**
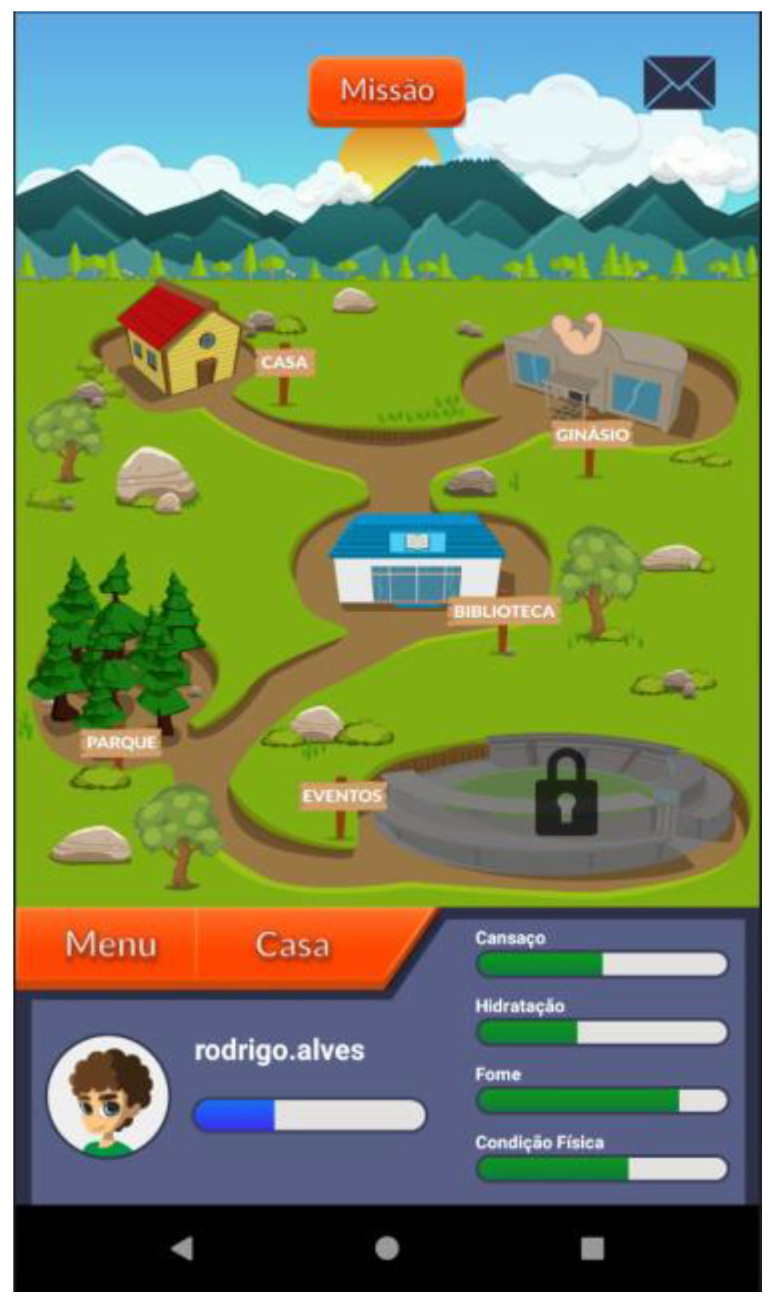
Dashboard—navigation map that holds: the user home (“casa”), the gym (“ginásio”), the library (“biblioteca”) and the park (“parque”)—affordance; legibility.

**Figure 4:**
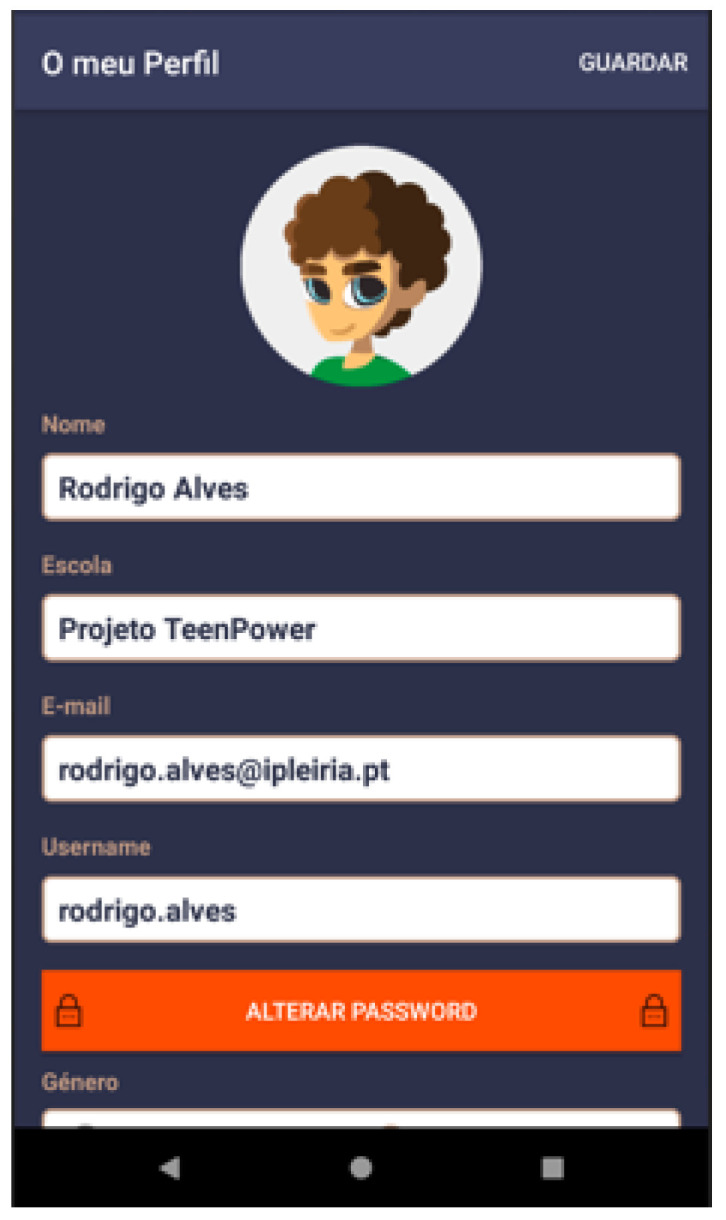
Profile—basic information such as name (“nome”) and school (“escola”)—storytelling.

**Figure 5:**
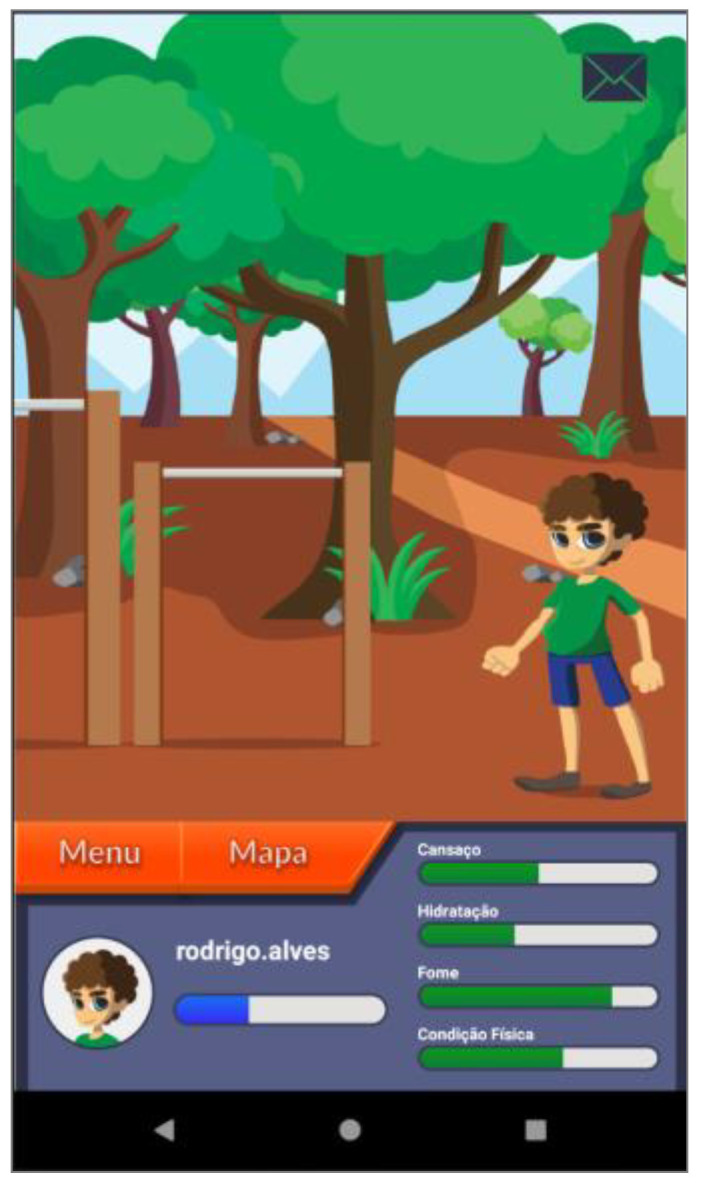
“Parque” (Park) section—presents the fatigue (“cansaço”); hydration (“hidratação”); hunger (“fome”) and physical condition (“condição física”)—affordance; consistency.

**Figure 6:**
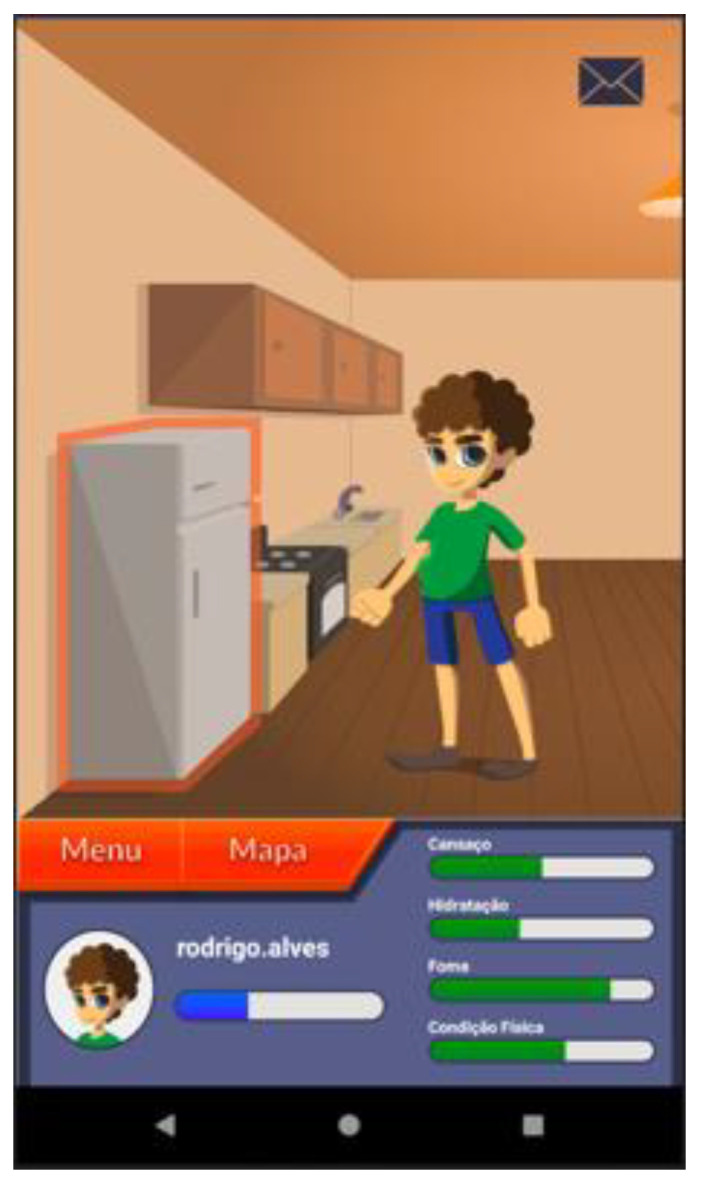
“Casa” Home section—presents the fatigue (“cansaço”); hydration (“hidratação”); hunger (“fome”) and physical condition (“condição física”)—affordance; consistency.

**Figure 7:**
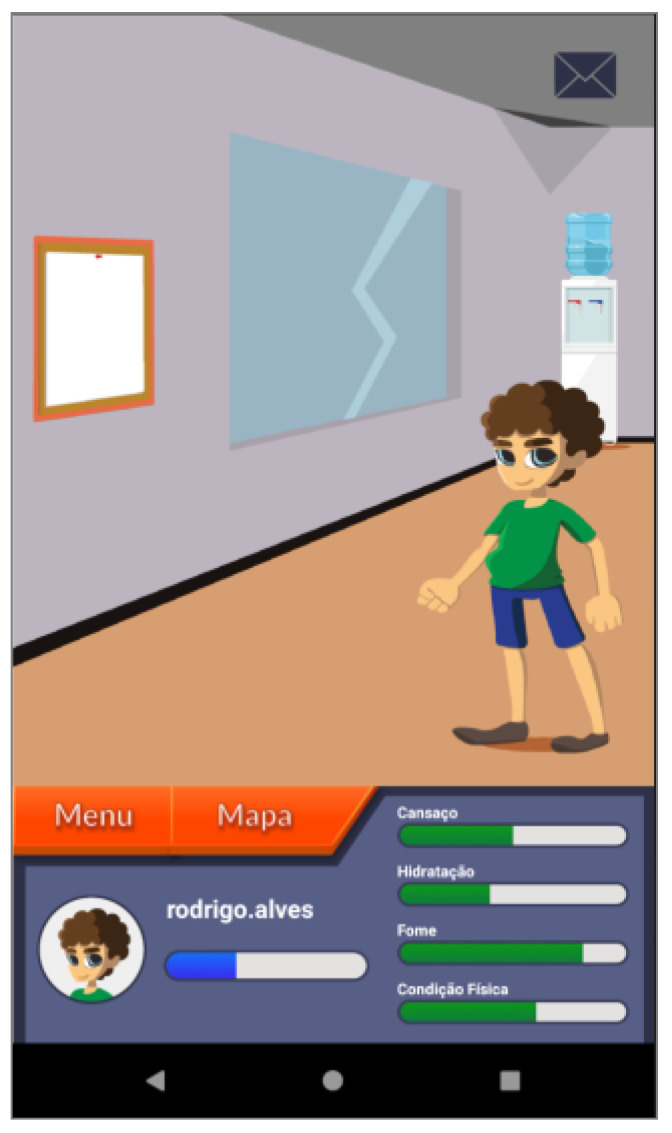
“Ginásio” Gym section—presents the fatigue (“cansaço”); hydration (“hidratação”); hunger (“fome”) and physical condition (“condição física”)—affordance; consistency.

**Figure 8:**
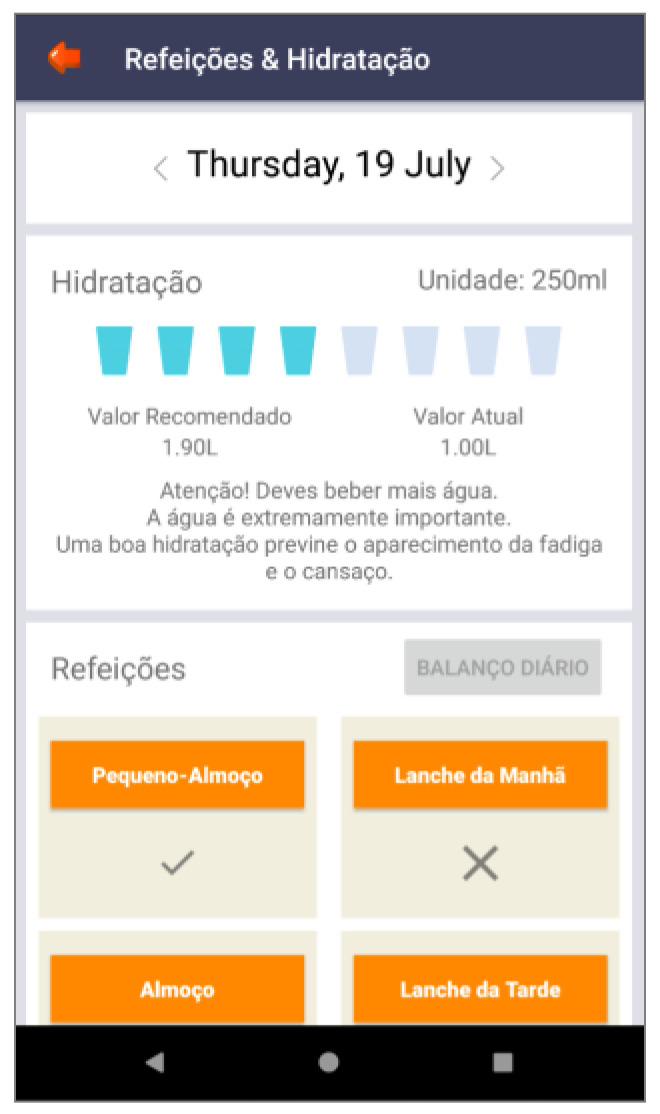
Back button—meals (“refeições” such as breakfast (“pequeno-almoço”), lunch (“almoço”) and hydration (“hidratação”) with water intake information and advice—mental model.

**Figure 9:**
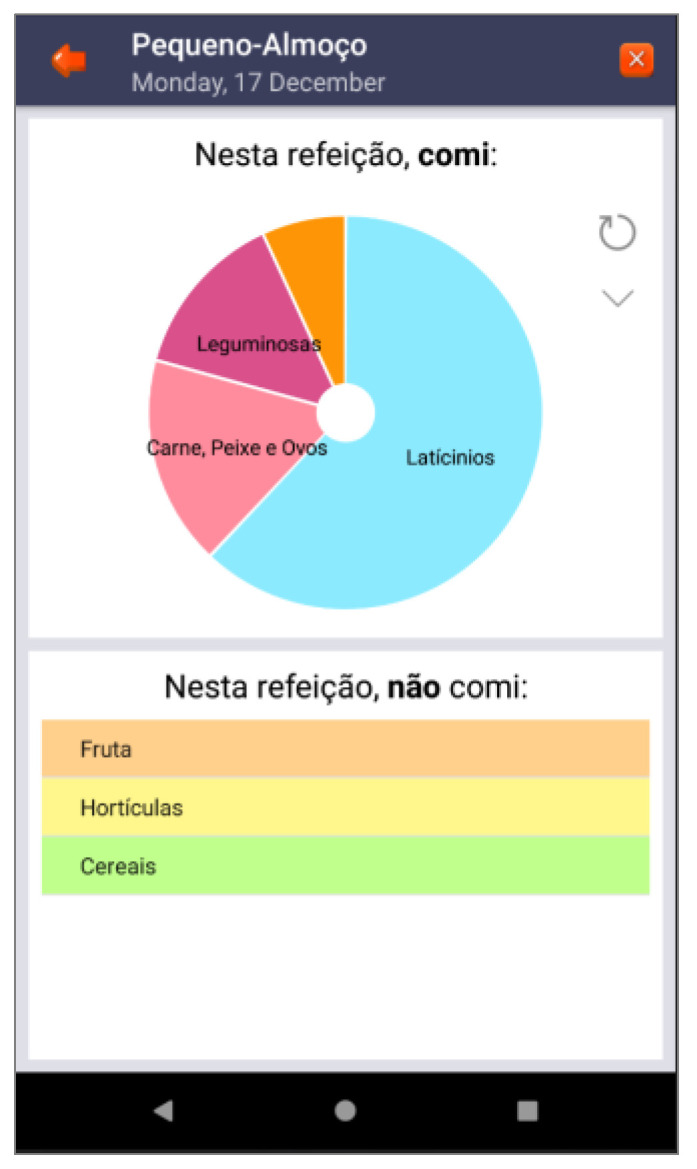
Back button—breakfast (“pequeno-almoço”) with the food intake presented in the wheel (meat = ”carne”, dairy = ”lacticínios”,…) and the major categories missed in that meal (fruit = ”fruta”, vegetables = ”hortícolas”, cereals = ”cereais”)—mental model.

**Figure 10:**
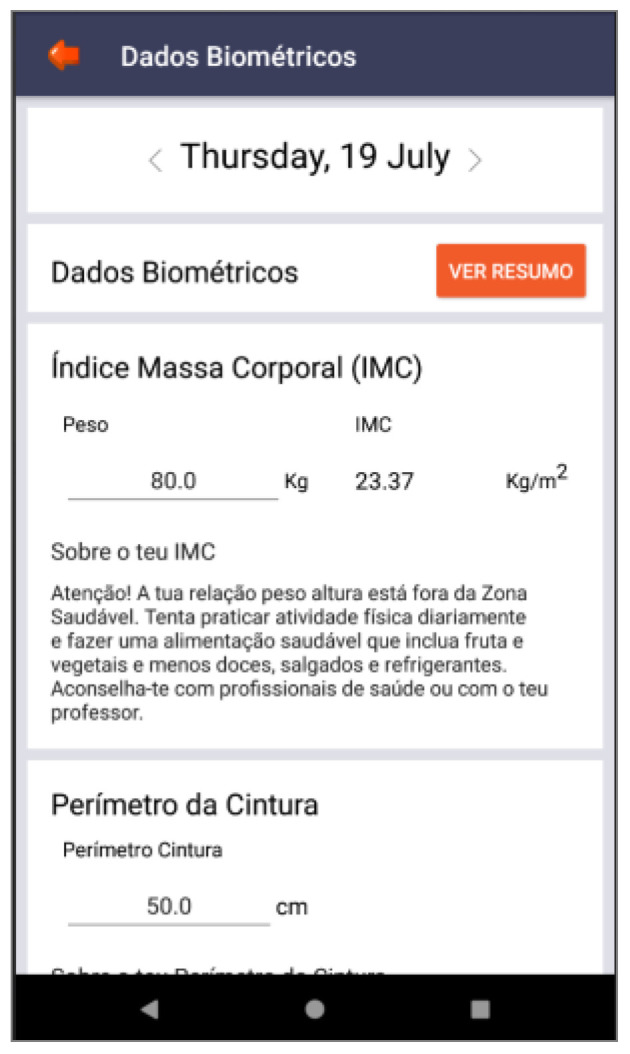
Back button—biomedical data (“dados biométricos”) with body mass index (BMI—“índice de massa corporal (IMC)), waist circumference (“perímetro da cintura”)—mental model.

**Table 1:** Strategy for pairing game elements design with health procedures.

Games Elements [1]	Universal Principles of Design [2]	Health Procedures	Figure Number
Three-dimensional virtual environments	Affordance	Integration of clinical procedures behind virtual environments	Figure 2, Figure 3, Figure 5, Figure 6 and Figure 7
Self-representation with avatars	Storytelling	Personal ID	Figure 4
Narrative context	Immersion; consistency	Common areas easily recognized by adolescents where they should complete requested healthy tasks	Figure 3
Feedback	Consistency; mental model	Back-office feedback that allows adolescents to remain committed to their health condition	Figure 3, Figure 8 and Figure 9
Ranks and levels	Feedback loop	Back-office feedback that motivates adolescents to continue playing the game	Figure 10
Competition under rules		Increase the motivation Fogg Behavior Model [3]	NA

## Data Availability

Not applicable.

## Appendix A. Record Sheet for the Observer

Record Sheet for the Observer—1 of *n* (number of tasks)

Name:				Area of Expertise:	
Gender:		Date of Birth:		Mobile Phone Model:	

start time:		end time:	

**Task #1** *Record at least 2 of the meals that you made yesterday. Can you tell me what was the percentage of vegetables that you consumed in those meals?*	
**Duration** How many seconds did the user take to complete?	
**Expected Path** Has the user followed the expected path?	
**[Expected Path]** Start—Big Avatar—Click on previous day arrow—Click on one meal (Breakfast or other)—Click on slices that I don’t eat (do that 2 times, 1 for each meal)—Click Back—Click on Daily Balance—See the percentage of vegetables.	
**Reaction** What were the user’s expressions and comments?	
**Errors** How many clicks in the wrong places?	
**User is LOST** What did the user expect the app would do?	

**Instructions**

To conduct the direct observation following a script you should consider three different roles:

(1) the volunteer—carried out by those that did not have any previous contact with the application. Explores the application by following instructions from the interviewer;

(2) the interviewer—carried out by those who had previous contact with the application. Guides the volunteer, launches the pre-defined tasks and also serves as a facilitator when the user is unable to advance according to the script. They also encourage the volunteer to talk out loud and share her/his opinions along the way;

(3) the observer (paired with an interviewer)—carried out by those who had previous contact with the application. Takes notes of every reaction and fills this record sheet (one per task) that implies registering data regarding several metrics, including task completion duration (time in seconds), number of wrong clicks, followed path (versus expected path), and the results of the “talk-aloud” method, amongst others.

**Notice**

It is important to mention that when a volunteer is unable to advance, the interviewer can help the volunteer by questioning her/him about her/his actions. This proves to be sufficient for the user to quickly realize what s/he is doing wrong.

## Appendix B. Final Questionnaire (for the User)

Final Questionnaire (for the Volunteer)

Name:				Area of Expertise:	
Gender:		Date of Birth:		Mobile Phone Model:	

How much time did you spend in the app until finding it easy to navigate? *Quanto tempo passou na aplicação até a considerar explorada?*	
Did you find the navigation flow simple and easy to use? If not, why? *Achou simples e fácil a navegação na aplicação? Se Não, Porquê?*	
Did you find the exercise monitoring area? Steps and calories? If yes, where? *Encontrou a zona de monitorização de atividade física? (Passos e calorias?) Se Sim, Onde?*	
Did you find the forum area? If yes, where? *Encontrou a zona do forúm? Sim, Onde?*	
Observations and Comments—*Observações e Comentários*	

## Appendix C. Post-Study System Usability Questionnaire (PSSUQ)—List of Items

Item 1—Overall, I am satisfied with how easy it is to use this system.

*Em geral, estou satisfeito com a facilidade de utilização deste Sistema.*

Item 2—It was simple to use this system.

*Este sistema foi simples de utilizar.*

Item 3—I could effectively complete the tasks and scenarios using this system.

*Consegui completar as tarefas e os cenários utilizando este sistema.*

Item 4—I was able to quickly complete the tasks and scenarios using this system.

*Consegui completar rapidamente as tarefas e cenários utilizando este sistema.*

Item 5—I was able to efficiently complete the tasks and scenarios using this system.

*Consegui completar as tarefas e os cenários com eficiência utilizando este sistema.*

Item 6—I felt comfortable using this system.

*Senti-me confortável a utilizar este sistema.*

Item 7—It was easy to learn to use this system.

*Foi fácil aprender a utilizar este sistema.*

Item 8—I believe I could quickly become productive using this system.

*Acredito que me tornaria rapidamente produtivo se utilizasse este sistema.*

Item 9—The system gave error messages that clearly told me how to fix problems.

*O sistema deu mensagens de erros que me indicaram claramente como resolver os problemas.*

Item 10—Whenever I made a mistake using the system, I could recover easily and quickly.

*Sempre que cometi um erro durante a utilização do sistema, consegui recuperar de forma fácil e rápida.*

Item 11—The information (such as online help, on-screen messages and other documentation) provided with this system was clear.

*A informação fornecida pelo sistema (como ajuda online, mensagens no ecrã ou outra documentação) foi clara.*

Item 12—It was easy to find the information I needed.

*Foi fácil encontrar a informação que precisava.*

Item 13—The information provided for the system was easy to understand.

*A informação fornecida pelo sistema foi fácil de entender.*

Item 14—The information was effective in helping me complete the tasks and scenarios.

*A informação foi eficaz para me ajudar a completar as tarefas e os cenários.*

Item 15—The organization of information on the system screens was clear.

*A organização da informação que o sistema transmitiu foi clara.*

Item 16—The interface of this system was pleasant.

*A interface do sistema foi agradável.*

Item 17—I liked using the interface of this system.

*Gostei de utilizar a interface deste sistema.*

Item 18—This system has all the functions and capabilities I expected it to have.

*Este sistema tem todas as funcionalidades e capacidades que eu esperava.*

Item 19—Overall, I am satisfied with this system.

*Em geral, estou satisfeito com este sistema.*
